# Multiplex PCR based screening for micro/partial deletions in the AZF region of Y-chromosome in severe oligozoospermic and azoospermic infertile men in Iran

**Published:** 2016-11

**Authors:** M. Burak Kaplan, Hasan Acar

**Affiliations:** *Department of Medical Genetics, Selcuk University, Medical Faculty, Konya, Turkey.*

**Dear Editor**

We interested in a very nice article by Motovali-Bashi *at al* entitled as “Multiplex PCR based screening for micro/partial deletions in the AZF region of Y-chromosome in severe oligozoospermic and azoospermic infertile men in Iran” (1). In this article, authors described the frequency of Y chromosome AZF microdeletions increased in subjects with severe spermatogenic failure and partial deletions of AZFc associated with spermatogenic failure. However, in figure 2 of the paper, sY1291 and sY1201 STS-PCR products on agarose gel were presented erroneously. This mistake does not match with the legend of figure 2. sY1201 and sY1291 PCR product sizes are 677 bp and 527 bp, respectively. According to STS-PCR product sizes, sY1201 should be corrected on the top PCR product band on the description of agarose gel. We would like to bring forward this mistake for your attention and for a possible correction.

## Conflict of interest

The authors declare that they have no conflicts of interests.

## Figures and Tables

**Figure 2:**
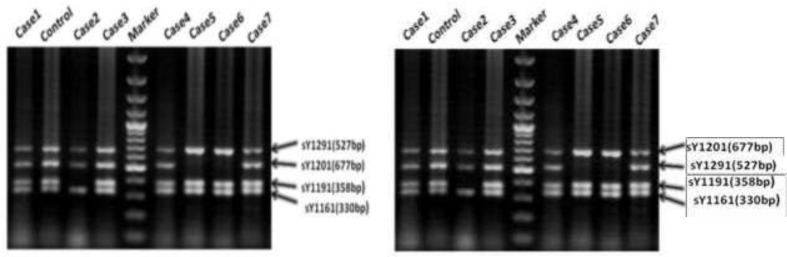
Detection of the gr/gr and b2/b3 partial deletions in the AZFc region using STS-PCR. Control: Normozoospermic man; Cases 2, 5, 6: The gr/gr deletions are defined by absence of sY1291 band and the presence of other STSs. Case 2: The b2/b3 deletions are defined by absence of sY1191 and the presence of other STSs. Electrophoresis conditions were constant 40 Voltage, including alternative current and 2% agarose gel. Marker: 100 bp DNA Marker.

## Editorial

Thanks to Dr. Burak Kaplan for his comments on Image (Left) which was already published in our journal issued 2015; 13: 563-570. Here, the corrected image by authors (right). The difference is about order of bands on image.

The corrected figure has been uploaded in our electronic version.

## References

[1] Motovali-Bashi M, Rezaei Z, Dehghanian F, Rezaei H (2015). Multiplex PCR based screening for micro/partial deletions in the AZF region of Y-chromosome in severe oligozoospermic and azoospermic infertile men in Iran. Iran J Reprod Med.

